# Plasma p-tau217, quantified by the fully automated LUMIPULSE G platform, outperforms p-tau181 in predicting amyloid pathology in cognitive complaints patients

**DOI:** 10.1038/s41598-025-34241-7

**Published:** 2026-01-15

**Authors:** Anuschka Silva-Spínola, João Durães, João Pedro Guedes de Oliveira, Miguel Tábuas-Pereira, Marisa Lima, Alexandre de Mendonça, Isabel Santana, Inês Baldeiras

**Affiliations:** 1 Center for Innovative Biomedicine and Biotechnology, University of Coimbra, Coimbra, 3004-504 Portugal; 2 Faculty of Medicine, University of Coimbra, Coimbra, 3000-548 Portugal; 3 Neurology Department, Coimbra University Hospital, Unidade Local de Saúde de Coimbra, Coimbra, 3004-561 Portugal; 4 Center for Research in Neuropsychology and Cognitive Behavioral Intervention, University of Coimbra, Coimbra, 3000-115 Portugal; 5 Faculty of Medicine, University of Lisbon, Lisbon, 1649-028 Portugal; 6 Neurochemistry Laboratory, Neurology Department, Coimbra University Hospital, Praceta Mota Pinto, Coimbra, 3004-561 Portugal

**Keywords:** Alzheimer’s disease, Biomarkers, Plasma, Tau protein, Dementia, Biomarkers, Neurology, Neuroscience

## Abstract

Plasma phosphorylated tau (p-tau) biomarkers offer a minimally invasive alternative for detecting Alzheimer’s disease (AD) pathology. We evaluated the diagnostic performance of plasma p-tau217 relative to p-tau181 using the fully automated LUMIPULSE platform. A total of 494 consecutively recruited patients with cognitive complaints from the Coimbra cohort were divided into exploratory and internal validation sets, and an external set of 100 well-characterized patients with mild cognitive impairment was obtained from Lisbon. Plasma biomarkers were assessed in relation to cerebrospinal fluid (CSF) AD biomarkers and evaluated their ability to detect amyloid pathology, defined by the CSF Aβ42/40 ratio. In the exploratory dataset, plasma p-tau217 showed stronger correlations with CSF biomarkers and higher accuracy in identifying amyloid positivity than p-tau181 (AUC = 0.90 vs. 0.81, *p* < 0.001). Age and blood urea nitrogen had minimal influence on p-tau217’s performance. Applying the Youden-derived single p-tau217 cutoff achieved an overall agreement of 88% with CSF-defined amyloid status across both validation sets. In addition, we evaluated a two-cutoff framework (95% sensitivity/specificity), which reduced misclassification and confined fewer than 30% of individuals to an intermediate, confirmatory-testing zone. These findings support the potential of plasma p-tau217 as a scalable blood-based biomarker that can aid triage and diagnostic decision-making in AD.

**Supplementary Information:**

The online version contains supplementary material available at 10.1038/s41598-025-34241-7.

## Introduction

The diagnosis of Alzheimer’s disease (AD) has undergone a paradigm shift with the integration of biomarkers that reflect the core neuropathological features of the disease. Cerebrospinal fluid (CSF) biomarkers, including amyloid beta (Aβ42 and Aβ42/Aβ40 ratio), phosphorylated tau 181 (p-tau181), and total tau (t-tau), have been recognized as central to research-based and clinical diagnostic frameworks^1^. More recently, advances in blood-based biomarkers (BBMs), particularly those measured in plasma, have shown considerable promise as minimally invasive tools for detecting AD pathology^2^. Recent diagnostic frameworks and research guidelines^3,4^ and the Alzheimer’s Association Clinical Practice Guideline on BBMs^5^ discuss how these markers may be incorporated into specialized care pathways.

Among these, plasma p-tau217 has emerged as a promising biomarker, showing stronger associations with amyloid and tau pathology^6^ and superior diagnostic accuracy compared to plasma p-tau181^7,8^. Notably, plasma p-tau217 levels correlate closely with amyloid- and tau-PET imaging and CSF biomarker profiles, reinforcing its utility as a minimally invasive surrogate of AD neuropathology^9,10^. These findings have been validated across diverse populations, including cohorts in Sweden, Spain, and Italy^9–12^. Additionally, it can be conceptualized according to its intended clinical role, either as a triage test, where high sensitivity is required to rule out AD pathology and positive cases proceed to CSF or amyloid PET confirmation, or as a confirmatory test, where both sensitivity and specificity must be high to establish the presence of AD pathology reliably^5^. However, translating these findings from research studies into real-world clinical settings poses challenges, including analytical variability, population heterogeneity, and comorbidities that may influence biomarker levels^13^. Therefore, more validation studies across broad clinical populations using standardized methodologies are critical.

Given the biological and clinical heterogeneity among individuals with cognitive impairment, evaluating the influence of systemic comorbidities on plasma biomarker levels is essential. Renal function has been studied as a potential modifier of circulating tau levels. Studies in patients with established chronic kidney disease (CKD) have shown that impaired renal clearance can elevate soluble tau species, including p-tau217 and p-tau181, although these effects are attenuated when using phosphorylated-to-unphosphorylated tau ratios^14^. However, these findings derive from patients with moderate-to-severe CKD, whereas age-related renal changes in routine clinical populations may be more subtle and heterogeneous. Routine laboratory markers, such as creatinine and blood urea nitrogen (BUN), can offer complementary insights, with BUN potentially detecting early renal changes not reflected by creatinine in older adults with reduced muscle mass^15^. Yet, BUN is also sensitive to hydration status, dietary protein intake, and certain medications^16^. Accordingly, we examined renal markers in our cohort as exploratory sensitivity analyses, rather than central confounders, to determine whether common physiological variability meaningfully influences p-tau217 in real-world clinical settings.

In addition, standardization and reproducibility are critical for the clinical adoption of BBMs. Studies from CSF biomarker research highlight the importance of using fully automated analytical platforms, which reduce pre-analytical and operator-dependent variability^17^. The recent launch of a plasma p-tau217 immunoassay on the fully automated LUMIPULSE platform represents a significant advancement, facilitating high-throughput with enhanced precision and reliability, and widespread implementation^11,12,18^.

In this study, we compared the diagnostic performance of plasma p-tau217 with that of plasma p-tau181 on the fully automated LUMIPULSE platform in a real-world clinical setting. We analyzed data from patients with cognitive complaints recruited from two distinct clinical contexts: one representing a broad and heterogeneous population from a tertiary hospital setting, and the other from a more selectively characterized memory clinic cohort. Biomarker cutoffs were established in an exploratory set and subsequently applied to both internal and external validation cohorts. We also examined the influence of demographic variables and common comorbidities, particularly renal function and metabolic status parameters, on plasma p-tau217 levels. Our primary goal was to determine whether plasma p-tau217 offers superior diagnostic performance compared to p-tau181 for detecting AD pathology and to evaluate its potential as a robust and scalable BBM for clinical use.

## Results

### Exploratory set characteristics

The exploratory set included 395 individuals from the Coimbra cohort, consecutively recruited at a tertiary neurology department for evaluation of cognitive complaints. The distribution of clinical diagnoses is presented in Table 1. No participant met clinical criteria for CKD based on medical history and available laboratory data. All individuals underwent CSF-AD biomarker assessment as part of their diagnostic workup, allowing classification of amyloid status (A) based on a validated, lab-specific CSF Aβ42/40 ratio (A positivity: <0.068)^19^. In this set (Table 1), 216 patients (55%) were classified as amyloid-negative (A-) and 179 as amyloid-positive (A+). The median age was 67.0 years, with A + individuals older than A- (70.0 vs. 64.5 years, *p* < 0.001). The proportion of females was similar between groups (A-: 56%, A+: 59%; *p* = 0.522).

**Table 1:** Baseline characteristics of the exploratory and validation sets stratified by amyloid status.

	Exploratory set (Coimbra)		Internal validation group (Coimbra)		External validation group (Lisbon)	
	A- (*n* = 216)	A+ (*n* = 179)	A- (*n* = 53)	A+ (*n* = 46)	A- (*n* = 39)	A+ (*n* = 61)
Demographic and clinical information						
Age, years	64.5 (59.0–70.0)	70.0 (64.0–74.0)^a^	67.0 (58.0–73.0)	71.0 (66.0-75.8)^b^	66.1 (56.6–72.0)	71.8 (67.1–75.0)^d^
Sex, % females (n)	56% (121)	59% (106)	77% (41)	63% (29)	54% (21)	59% (36)
Diagnosis, % (SCD/MCI/ Dementia/Other)	9/60/29/2	1/50/49/0^a^	8/68/24/0	2/43/55/0^b^	0/100/0/0	0/100/0/0
CSF and blood biomarker concentration						
CSF Aβ42/40 ratio	0.108 (0.098–0.114)	0.051 (0.043–0.057)^a^	0.099 (0.086–0.107)	0.053 (0.045–0.058)^c^	0.090 (0.080–0.102)	0.050 (0.040–0.060)^e^
CSF p-tau181, pg/mL	32.3 (23.3–43.1)	93.5 (61.7–123.0)^a^	33.3 (24.3–48.2)	93.3 (67.1-129.1)^c^	39.0 (29.5–46.5)	84.0 (61.0-107.0)^e^
CSF t-tau, pg/mL	246.5 (185.8–322.0)	567.0 (379.5-807.5)^a^	255.0 (169.0-332.0)	578.5 (462.3–750.0)^c^	295.0 (212.0-395.0)	594.0 (441.0-788.0)^e^
AT(N) scheme, % (n): Normal	79% (170)	0% (0)^a^	74% (39)	0% (0)^c^	74% (29)	0% (0)^e^
AD continuum	0% (0)	100% (179)	0% (0)	100% (46)	0% (0)	100% (0)
Non-AD pathology	21% (46)	0% (0)	26% (14)	0% (0)	26% (10)	0% (0)
Plasma p-tau181, pg/mL	1.12 (0.81–1.51)	2.22 (1.63–2.93)^a^	-	-	-	-
Plasma p-tau217, pg/mL	0.10 (0.07–0.15)	0.56 (0.28–0.87)^a^	0.10 (0.08–0.14)	0.51 (0.34–0.77)^c^	0.10 (0.08–0.16)	0.58 (0.37–0.94)^e^
Blood glucose, mg/dL	93.0 (84.0-111.5)	93.0 (85.0-108.0)	-	-	-	-
Blood creatinine, mg/dL	0.76 (0.68–0.88)	0.77 (0.70–0.86)	-	-	-	-
Blood urea nitrogen, mg/dL	16.0 (13.7–19.7)	17.2 (13.6–20.6)	-	-	-	-

Data is presented as median (25th -75th percentiles) or as a percentage. The table compares three groups: the exploratory set (Coimbra, *n* = 395), the internal validation set (Coimbra, *n* = 99), and the external validation set (Lisbon, *n* = 100), with group comparisons performed separately by amyloid status (A). Amyloid status was determined using validated laboratory-specific CSF Aβ42/40 cutoffs (< 0.068 for Coimbra and < 0.072 for Lisbon) and/or positive amyloid PET (Lisbon). Continuous variables were compared using the Wilcoxon rank-sum test and categorical variables using Pearson’s chi-squared test. Superscripts indicate statistically significant comparisons: (a) exploratory set *p* < 0.001; (b) internal validation *p* = 0.011; (c) internal validation *p* < 0.001; (d) external validation *p* = 0.001; (e) external validation < 0.001; variables without superscripts were not significant (*p* > 0.05). CSF biomarkers were measured using the LUMIPULSE G600II platform in Coimbra and the LUMIPULSE G1200 in Lisbon, whereas all plasma biomarkers were measured on the LUMIPULSE G600II of Coimbra. Aβ = amyloid beta; CSF = cerebrospinal fluid; MCI = mild cognitive impairment; n = count or number of individuals per variable; p-tau181 = phosphorylated Tau protein in position 181; p-tau217 = phosphorylated Tau protein in position 217; SCD = Subjective cognitive decline; t-tau = total Tau protein.

CSF biomarker concentrations differed between groups by amyloid status. The median CSF Aβ42/40 ratio was lower in A+ (0.051) compared to A- (0.108; *p* < 0.001) participants. Similarly, CSF p-tau181 and t-tau concentrations were higher in the A + group (p-tau181: 93.5 vs. 32.3 pg/mL; t-tau: 567.0 vs. 246.5 pg/mL; both *p* < 0.001). These results aligned with the AT(N) scheme (T positivity: CSF p-tau181 > 51.2 pg/mL, and N positivity: CSF t-tau > 354 pg/mL^19^: among A- individuals, 79% were classified as having normal biomarker profiles and 21% as non-AD pathological change. In contrast, A + individuals were predominantly classified within the A+T+ category (84%). As depicted in Table 1, no differences were observed in blood glucose, creatinine, or urea nitrogen between the two groups.

### Plasma p-tau181 and p-tau217 levels by amyloid and/or tau status

Plasma concentrations of p-tau181 and p-tau217 were compared between the A- and A + groups defined above. As depicted in Table 1, plasma p-tau181 levels were nearly double in A + compared to A- individuals (2.22 vs. 1.12 pg/mL; *p* < 0.001). Plasma p-tau217 showed an almost 6-fold increase, with median values of 0.56 pg/mL in A + and 0.10 pg/mL in A- individuals (*p* < 0.001). To ensure these differences were not driven by the older age of the A + group, we performed an age-adjusted analysis by residualizing plasma p-tau values for age. Both biomarkers remained higher in A + than in A- individuals after adjustment (*p* < 0.0001 for both).

**Fig. 1:**
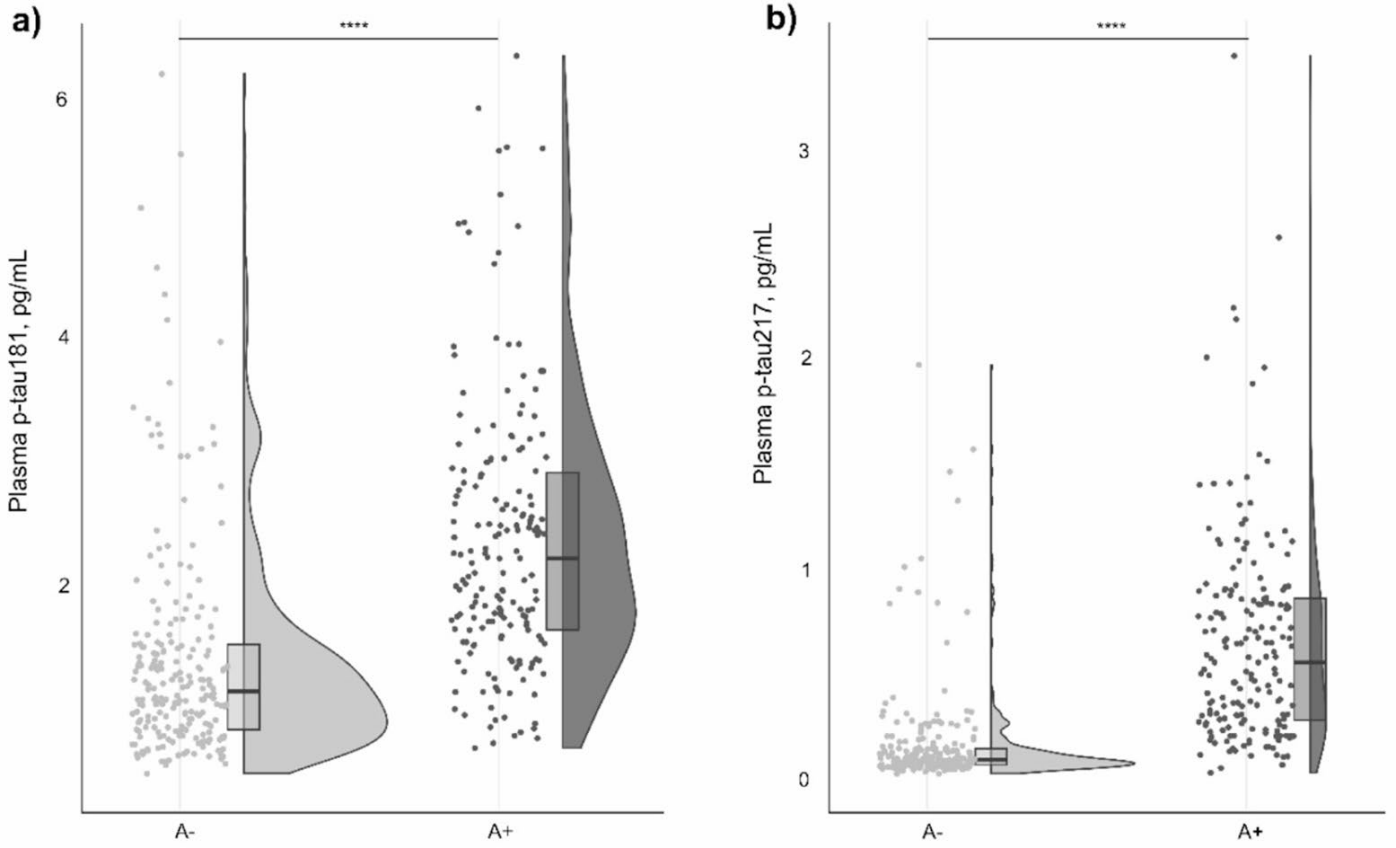
Distribution of plasma p-tau181 and p-tau217 concentrations according to amyloid status in the exploratory set. Scatter plots, box plots, and violin plots are combined to display plasma p-tau181 (panel **a**) and p-tau217 (panel **b**) concentrations stratified by amyloid status. Amyloid classification was based on a validated, laboratory-specific CSF Aβ42/40 cutoff for the Coimbra cohort (A + < 0.068). Violin plots show data density, while box plots indicate median and interquartile range, and Individual data points are plotted as dots. Statistical significance: **** p-value < 0.0001.

Figure 1 shows that plasma p-tau217 has a narrower distribution in A- individuals, improving separation from the A + group relative to p-tau181. These patterns were reflected in the effect size estimates: plasma p-tau181 showed a Cliff’s delta of -0.61 (95%CI: -0.69 to -0.51), while plasma p-tau217 demonstrated a larger effect size of -0.80 (95%CI: -0.86 to -0.73). Effect sizes differed (∆=-0.16, *p* < 0.001 with a bootstrap of 1000 replicates), confirming the superior discriminative performance of plasma p-tau217.

As we examined plasma p-tau181 and p-tau217 concentrations across CSF-derived AT classification groups in the exploratory set (Supplementary Tables 1 and 2), we observed that individuals with normal amyloid and tau status (A-T-, *n* = 186) had the lowest plasma levels of both biomarkers (median p-tau181: 1.06 pg/mL, p-tau217: 0.09 pg/mL). Plasma levels were modestly elevated in those with isolated tau pathology (A-T+, *n* = 30) or isolated amyloid pathology (A+T-, *n* = 29), and were highest in the A+T+ group (*n* = 150), reaching 2.40 pg/mL for p-tau181 and 0.65 pg/mL for p-tau217. Group-wise comparisons revealed that both biomarkers effectively distinguished A-T- from the other AT categories (Supplementary Table 1). For plasma p-tau181, differences were observed between A-T- and A-T+ (*p* = 0.002), A+T- (*p* < 0.001), and A+T+ (*p* < 0.001), as well as between A-T + and A+T+ (*p* < 0.001), and A+T- and A+T+ (*p* < 0.001). Only A-T + and A+T- did not reach statistical significance (*p* = 0.055). Similarly, plasma p-tau217 levels differentiated A-T- from A-T+ (*p* < 0.001), A+T- (*p* = 0.003), and A+T+ (*p* < 0.001), and also distinguished A-T+ from A+T+ (*p* < 0.001), while showing no difference between A-T+ and A+T- (*p* = 0.96).

In the exploratory set, plasma p-tau217 and p-tau181 were strongly correlated (Spearman’s ρ = 0.84, *p* < 0.001). However, correlation analyses of core CSF biomarkers revealed stronger associations with p-tau217, consistent with previous reports^11^. Specifically, plasma p-tau217 levels were more strongly correlated with CSF Aβ42/40 ratio (ρ = -0.62), CSF p-tau181 (ρ = 0.72), and CSF t-tau (ρ = 0.64) than plasma p-tau181 (ρ = -0.46, 0.60, and 0.52, respectively; all *p* < 0.001). Steiger’s Z-tests confirmed these differences for dependent overlapping correlations: z = -6.78 for CSF Aβ42/40 ratio, z = 5.79 for CSF p-tau181, and z = 5.27 for CSF t-tau (all *p* < 0.001), supporting an agreement of plasma p-tau217 with central AD biomarkers.

### Diagnostic performance of plasma p-tau biomarkers

**Fig. 2:**
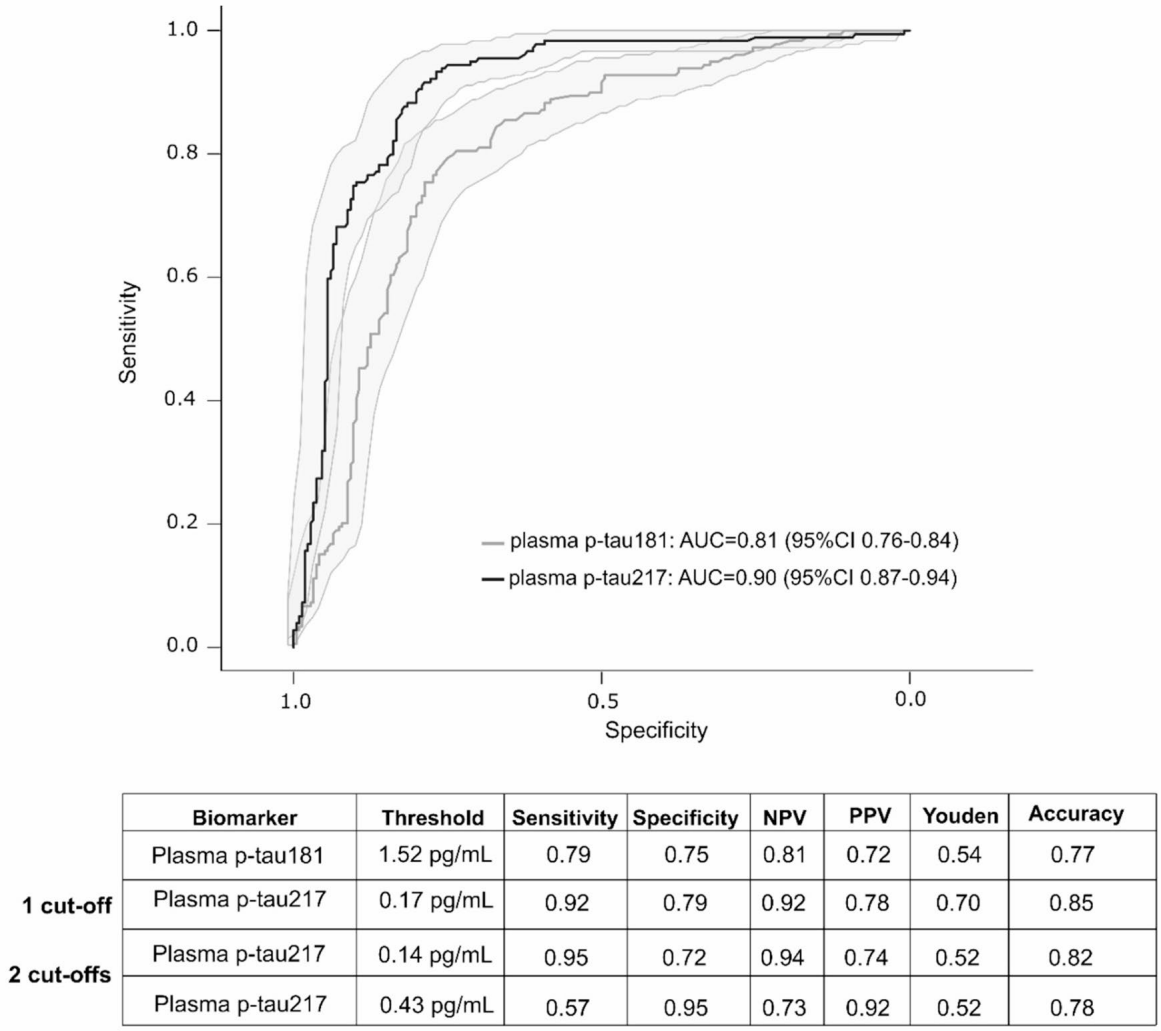
ROC curve analysis of plasma p-tau181 and p-tau217 for predicting amyloid status in the exploratory set. Receiver operating characteristic (ROC) curves illustrate the diagnostic performance of plasma p-tau181 (grey) and p-tau217 (black) in distinguishing amyloid-positivity (A+, *n* = 179) from amyloid-negative (A-, *n* = 216) individuals within the exploratory set (*n* = 395), using CSF Aβ42/40 < 0.068 as the reference standard. The area under the curve (AUC) is presented with 95% confidence intervals (CI). The table below the graph presents performance metrics for each analyte, including Youden-derived optimal thresholds for plasma p-tau181 and p-tau217 (single cutoff approach), the high sensitivity (95%) and high specificity (95%) threshold used in the 2-cutoff approach for p-tau217, as well as sensitivity, specificity, positive predictive value (PPV), negative predictive value (NPV), Youden’s index, and overall accuracy.

Using the exploratory set (*n* = 395), ROC analyses evaluated the ability of plasma biomarkers to discriminate amyloid status (A + vs. A-) as defined by the CSF Aβ42/40 ratio (Fig. 2). Plasma p-tau181 demonstrated an AUC of 0.81 (95%CI: 0.76–0.84), with a Youden’s based optimal threshold of 1.52 pg/mL. At this cutoff, sensitivity was 0.79, specificity 0.75, NPV 0.81, PPV 0.72, Youden’s index 0.54, and overall classification accuracy 0.77. In contrast, plasma p-tau217 achieved a higher AUC of 0.90 (95%CI: 0.87–0.94), with a Youden’s based optimal threshold of 0.17 pg/mL. This value yielded a sensitivity of 0.92, specificity of 0.79, NPV of 0.92, PPV of 0.78, Youden’s index of 0.70, and classification accuracy of 0.85. It is essential to note that the 95% CIs for the AUCs did not overlap, and the comparison of the two ROC curves favored plasma p-tau217 (*p* < 0.001). According to the recent BBM Clinical Practice Guideline^5^, this single cutoff performance for plasma p-tau217 meets criteria for an “accurate triage-level” test, achieving > 90% sensitivity and > 75% specificity.

To further assess marker performance in a more strictly defined pathological comparison, supplementary ROC analysis was conducted using A-T- (*n* = 186) versus A+T+ (*n* = 150) patients (Supplementary Table 2 and Supplementary Fig. 1). Results again favored p-tau217: for p-tau181, the AUC was 0.86 (95%CI: 0.82–0.90), sensitivity 0.85, specificity 0.77, and accuracy 0.81. For p-tau217, the AUC increased to 0.95 (95%CI: 0.92–0.97), with a sensitivity of 0.95, specificity of 0.82, and accuracy of 0.88. These curves differed (*p* < 0.001), with no overlap between the 95% confidence intervals.

Additionally, we compared the diagnostic performance of plasma p-tau217 across the two classification systems, based solely on amyloid status (A- vs. A+) and combined AT status (A-T- vs. A+T+). P-tau217’s AUC differed between the two frameworks (*p* = 0.03), suggesting improved discriminative ability for plasma p-tau217 when both amyloid and tau pathology are considered.

Lastly, we evaluated a two-cutoff approach, with high-sensitivity and high-specificity thresholds for plasma p-tau217. A lower cutoff of 0.14 pg/mL maximized sensitivity (0.95) and is suitable for “rule-out” triage use. In contrast, a higher cutoff of 0.43 pg/mL maximizes specificity and is appropriate for “rule-in” confirmation, as recommended by the BBM Clinical Practice Guideline^5^. More detailed performance metrics are shown in Fig. 2.

### Multivariate prediction model for amyloid status

To examine whether demographic or systemic variables improved diagnostic classification beyond plasma p-tau217 alone, we calculated a logistic regression model including p-tau217, age, and BUN as predictors using the exploratory set (*n* = 366). Plasma p-tau217 was the strongest independent predictor of amyloid positivity (OR = 100.4, 95%CI: 36.4-317.3, *p* < 0.001). Age was associated with amyloid positivity (OR = 1.1, 95%CI: 1.1–1.2, *p* < 0.001), whereas BUN showed an inverse association (OR = 0.95, 95%CI: 0.9-1.0, *p* = 0.046).

The overall model reached an AUC of 0.90 (95%CI: 0.86–0.93), comparable to plasma p-tau217 alone (AUC = 0.90, 95%CI: 0.87–0.94) and superior to plasma p-tau181 (AUC = 0.81, 95%CI: 0.76–0.84). However, a comparison of the ROC curves for plasma p-tau217 alone and the regression model showed no difference (*p* = 0.429), suggesting that plasma p-tau217 alone carried most of the discriminative power for amyloid status, while the covariates contributed minimally (Supplementary Fig. 2).

Other laboratory blood (glucose and creatinine), demographic variables, and their various combinations (including BUN and age), were evaluated as potential predictors of amyloid status. These variables were investigated individually and in multivariate logistic regression models; however, none of the candidates reached statistical significance or improved the models’ predictive performance.

Full regression results for both the logistic model predicting amyloid positivity and the linear models assessing determinants of plasma p-tau217 (below) are reported in Supplementary Table 3.

### Assessment of plasma p-tau217 determinants

We next evaluated whether common laboratory markers of renal or metabolic status influenced plasma p-tau217 concentrations. In a linear regression model including BUN and age, the model (F-statistic = 4.75, *p* = 0.009) explained only a small proportion of the variance (adjusted R^2^ = 0.020). BUN showed a small association with plasma p-tau217 (β = 0.009, *p* = 0.03), while age trended toward statistical significance (β = 0.005, *p* = 0.09). Given the minimal variance explained, these associations are unlikely to be clinically meaningful.

To contextualize this finding, we repeated the linear regression analysis, replacing BUN with creatinine. This model was not significant (F-statistic = 2.19, *p* = 0.11; adjusted R^2^ = 0.007), and creatinine showed no association with p-tau217 (β = 0.03, *p* = 0.24), while age demonstrated a small effect (β = 0.006, *p* = 0.04). Together, these sensitivity analyses indicate that routine renal markers have minimal impact on plasma p-tau217 levels in this cohort.

Other routinely collected laboratory measures and demographic variables were examined individually and in multivariable models, and none were significantly associated with plasma p-tau217.

### Validation of plasma p-tau217 cutoffs

Given its superior classification performance in the exploratory set, plasma p-tau217 was selected for further evaluation in two independent validation sets: an internal set from the Coimbra cohort (*n* = 99) and an external set from the Lisbon cohort (*n* = 100). Amyloid status was determined using the validated CSF Aβ42/40 cutoffs specific to each cohort (internal: <0.068; external: <0.072), and, in the external, by amyloid PET when available, as a confirmatory criterion. Descriptive statistics for each validation set are provided in Table 1.

**Fig. 3:**
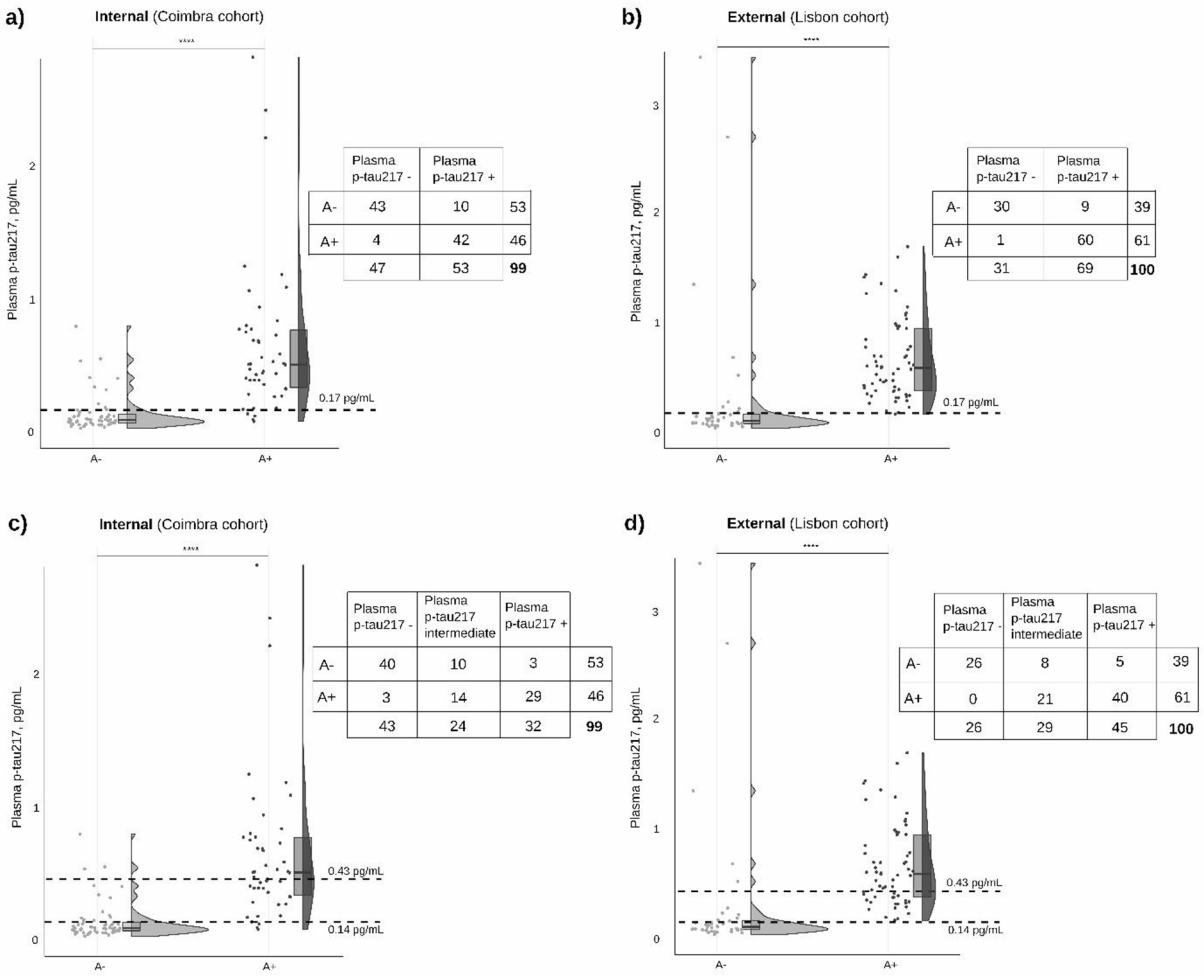
Distribution of plasma p-tau217 concentrations according to amyloid status in the validation sets (Coimbra and Lisbon cohorts). Scatter plots, box plots, and violin plots are combined to display the distribution of plasma p-tau217 according to amyloid-negative (A-) or amyloid-positive (A+) status, based on a validated laboratory-specific CSF Aβ42/40 cutoff, in two independent groups. Panels (**a**) and (**c**) are the internal (Coimbra) cohort (*n* = 99) and (**b**) and (**d**) the external (Lisbon) cohort (*n* = 100). Amyloid status was determined using each cohort’s validated CSF Aβ42/40 cutoff (internal: <0.068; external: <0.072) and amyloid PET when available (external). For panels (**a**) and (**b**), a horizontal dashed line marks the threshold of 0.17 pg/mL as the Youden-derived optimal threshold (single cutoff approach), obtained from the exploratory set (*n* = 395); and in panels (**c**) and (**d**), horizontal dashed line marks illustrate classification using a two cutoff approach: lower cutoff (95% sensitivity) of 0.14 pg/mL and upper cutoff (95% specificity) of 0.43 pg/mL. Violin plots illustrate the full data distribution and density, while box plots indicate median and interquartile range. Individual data points are shown as dots. Statistical significance is presented as: **** p-value < 0.0001. Classification matrices are displayed on the right-hand side of each panel.

Figure 3a,b display plasma p-tau217 concentrations using the Youden-derived threshold of 0.17 pg/mL. In the internal validation set, 43 of 53 A- individuals (81%) and 42 of 46 A + patients (91%) were correctly classified, resulting in an overall percentage agreement (OPA) of 86%. In the external validation set, classification accuracy was even higher: 30 of 39 A- (77%) and 60 of 61 A + individuals (98%) were correctly classified, corresponding to an OPA of 90%. Across both cohorts, the combined OPA was 88%.

Additionally, a ROC analysis of the combined validation set (*n* = 199; 92 A- and 107 A+) granted an AUC of 0.91 (95%CI: 0.86–0.96), closely matching the performance observed in the exploratory set. ROC curves did not differ (*p* = 0.81), indicating that the biomarker’s diagnostic accuracy was consistently maintained across independent samples.

To align with clinical recommendations advocating dual thresholds for triaging and confirmation^5^, we applied two cutoffs: a lower one with high sensitivity at 0.14 pg/mL and a higher one with high specificity at 0.43 pg/mL. Figure 3c,d illustrate the three resulting categories: p-tau217 negative (≤ 0.14 pg/mL), intermediate (0.14–0.43 pg/mL), and p-tau217 positive (≥ 0.43 pg/mL). In the internal set, 40/53 A- individuals (75%) fell below the low cutoff, and 29/46 A + individuals (63%) exceeded the high cutoff. Only 24% of the sample fell into the intermediate zone. In the external set, 26/39 A- individuals (67%) were below the low cutoff, 40/61 A + individuals (66%) were above the high cutoff, and 29% were intermediate. These rule-out and rule-in zones increased diagnostic certainty, decreasing the misclassification risk to 5–6%, while the intermediate range captures ambiguous cases appropriate for confirmatory testing (less than 30%).

## Discussion

In this study, we validated the diagnostic performance of the fully automated plasma p-tau217 assay (LUMIPULSE platform) across two real-world clinical cohorts encompassing a broad spectrum of cognitive decline, ranging from subjective complaints to mild cognitive impairment and dementia. Plasma p-tau217 levels showed moderate to strong correlations with core CSF AD biomarkers, particularly CSF p-tau181 and CSF Aβ42/40, and outperformed plasma p-tau181 in correlation strength and diagnostic accuracy. Group-wise analyses further confirmed that plasma p-tau217 effectively distinguished between CSF AT-defined pathology groups, especially among those with co-occurring amyloid and tau pathology. In the exploratory set, p-tau217 showed higher discriminative performance for amyloid positivity based on the CSF Aβ42/40 ratio (AUC = 0.90) than p-tau181 (AUC = 0.81), with non-overlapping 95% CIs and ROC curves that differed (*p* < 0.001). The optimal single-cutoff demonstrated robust performance in both validation sets, achieving a combined OPA of 88%. Applying a two-cutoff framework decreased misclassification to 5–6% and the intermediate zone was 24–29%, consistent with guideline-based recommendations. While age and renal function (BUN) showed small associations with p-tau217 levels, together they explained only 2% of variance (adjusted R^2^ = 0.020), and adding them to p-tau217 did not improve discrimination for amyloid positivity (same AUC of 0.90 for p-tau217 alone and the multivariate model), indicating that the biomarker itself carries almost all the predictive information.

In our study, plasma p-tau217 consistently outperformed plasma p-tau181 across multiple aspects, including correlation with core CSF AD biomarkers, fold-change across CSF-defined amyloid status and AT classification groups, and overall diagnostic accuracy. These results align with previous findings^11,20–22^. Although plasma p-tau isoforms typically exhibit a narrower dynamic range, we observed a fold change for plasma p-tau181 similar to that in prior studies^11,23^, and an even greater change for plasma p-tau217 (6-fold vs. 4-fold). Furthermore, the strength of association between the two plasma isoforms was higher for p-tau217, as reflected by a stronger correlation than in a previous study^11^ (ρ = 0.85 vs. 0.75), supporting the assay’s validity.

The moderate to strong correlations observed between plasma p-tau217 and core CSF AD biomarkers (ρ = -0.62 for CSF Aβ42/40, ρ = 0.72 for CSF p-tau181, and ρ = 0.64 for CSF t-tau) further support its biological validity. Slightly higher yet comparable correlation values have been reported using the LUMIPULSE platform in independent cohorts^11,12,18^. These findings reinforce the close concordance between plasma and CSF amyloid and tau pathology measures of amyloid and tau pathology. Neuropathological studies further validate this, which demonstrated a stronger association between plasma p-tau217 levels and the presence of amyloid plaques and tau tangles, relative to plasma p-tau181^6^.

Our findings align with the accumulating evidence demonstrating the strong diagnostic performance of plasma p-tau217, as measured by the fully automated LUMIPULSE platform^11,12,18,22–26^. In our cohort, plasma p-tau217 gave an AUC of 0.90 for discriminating amyloid positivity based on CSF Aβ42/40, within the reported range of 0.89 to 0.94^11,12,18,22–26^. Lower AUCs were typically observed in more heterogeneous clinical populations, while higher values were found in well-characterized research cohorts, supporting the robustness of plasma p-tau217 in real-world settings. Furthermore, within the AT(N) framework, p-tau217 achieved an AUC of 0.95 for identifying A+T+ individuals, exceeding the previously reported value on the same platform and highlighting its utility for biologically staging AD pathology^27^.

Across published work using the LUMIPULSE platform, proposed plasma p-tau217 thresholds demonstrate convergence despite differences in reference standards, cohort characteristics, and analytical strategies. Single cutoffs reported in the literature range from 0.095 to 0.274 pg/mL, with higher thresholds typically observed in studies referencing PET or CSF ratios incorporating tau^12,28^, and lower thresholds in clinically heterogeneous samples^29^. Our Youden-derived cutoff (0.17 pg/mL) lies at the lower end and closely matches values reported in studies using the CSF Aβ42/40 ratio^11,24,26^. This concordance highlights the methodological consistency of our findings and the robustness of p-tau217 performance across diverse real-world settings.

Furthermore, our single Youden-derived threshold (0.17 pg/mL; sensitivity 0.92, specificity 0.79) fulfils the Alzheimer’s Association criterion^5^ for an “accurate triage-level” BBM test. In settings where confirmatory CSF/PET is not immediately available, such as primary care or general neurology clinics, this single cutoff could be used to guide referral and further biomarker work-up. In individuals with cognitive complaints and clinical suspicion of AD, a plasma p-tau217 value below 0.17 pg/mL would make underlying AD pathology unlikely and support local management and clinical follow-up. In contrast, values at or above this value would indicate an increased likelihood of AD and favor referral to specialized memory clinics for confirmatory testing and treatment planning.

Two-cutoff frameworks, as recommended in the Alzheimer’s Association BBM Clinical Practice Guideline, were also evaluated. Published lower (rule-out) thresholds range from 0.14 to 0.23 pg/mL and upper (rule-in) thresholds from 0.34 to 0.56 pg/mL, with sensitivities and specificities above 95% across studies^11,12,23,28^. Our thresholds (0.14 and 0.43 pg/mL) fall within these ranges and produced comparable intermediate zone (≈ 24–29%), similar to values reported in the literature (typically 12–30%). Misclassifications with this approach were limited to (≈ 5–6%), consistent with prior reports showing that dual thresholds increase diagnostic certainty while reserving a modest proportion of cases for confirmatory CSF or PET testing^30^.

Clinically, our two-cutoff approach enables a pragmatic algorithm in specialized care, in line with recent guidelines and workflow proposals for blood p-tau-based testing^5,30,31^. In patients referred to memory clinics with clinical suspicion of AD, plasma p-tau217 ≤ 0.14 pg/mL defines a rule-out zone, in which the likelihood of underlying AD pathology is low and alternative diagnoses and clinical follow-up are advisable. Values ≥ 0.43 pg/mL define the rule-in zone, indicating a high likelihood of AD-continuum pathology; in these patients, clinicians may proceed directly to AD-focused management and reserve CSF/PET for cases with atypical clinical presentations or treatment-eligibility reservations. Results in the intermediate zone should trigger confirmatory testing with CSF or amyloid PET, or repeat BBM testing, depending on clinical urgency.

The single-cutoff approach is well-suited for triage across care levels, while the two-cutoff framework offers a more granular strategy in specialized settings. Always accounting for the fact that the BBM test results should always be interpreted within the clinical context^5^.

The inclusion of two distinct clinical cohorts for validation allowed for a robust evaluation of plasma p-tau217 across varied patient populations. The external validation set, derived from a private clinical practice, represents a more urban, highly educated, well-characterized, and preselected group, subject to a detailed neuropsychological and biomarker evaluation. In contrast, the exploratory and internal validation sets are drawn from a more clinically heterogeneous group, comprising general neurology consultations at a tertiary public hospital. Also, it reflects a more rural and less formally educated population^32^. This contrast offers a broader epidemiological perspective and strengthens the generalizability of our findings. The reproducibility of p-tau217 performance across these two settings highlights its potential utility as a scalable diagnostic tool across diverse healthcare contexts.

An important aspect of our evaluation was the potential influence of systemic factors on plasma p-tau217, an area that remains poorly understood. Previous studies have reported higher plasma p-tau217 in individuals with reduced kidney function^26^, whereas others found that age, not renal dysfunction, was the key contributor^12^. In our cohort, which did not include patients with established CKD, BUN showed a small statistical association with p-tau217 in sensitivity analyses. Still, the variance explained was minimal, and the effect did not replicate when creatinine was examined instead. Notably, the magnitude of these associations was far smaller than those previously reported in CKD-based studies^14^. Given differences in renal markers, analytical platforms, and disease severity across studies, our findings suggest that routine renal variability is unlikely to exert a clinically meaningful influence on plasma p-tau217 in typical diagnostic populations.

Emerging strategies, such as combining p-tau217 with Aβ42 or computing %p-tau217 (p-tau217/non-p-tau217 × 100) via mass spectrometry, have demonstrated improved diagnostic performance in research cohorts^12,25,26^. However, these gains are often modest. For example, only a slight increase in diagnostic accuracy has been reported when comparing p-tau217 alone (AUC = 0.94) with the p-tau217/Aβ42 ratio (AUC = 0.96)^26^. Because ratio-based approaches require additional assays, greater analytical complexity, and higher costs, their added value may be justified in settings requiring the highest degree of diagnostic certainty. In contrast, our findings show that plasma p-tau217 alone, when measured using a single automated assay, achieves strong accuracy and supports both single- and two-cutoff clinical workflows. This makes p-tau217 a more practical, scalable, and resource-efficient option for routine diagnostic implementation.

This study offers several novel strengths. First, we validated plasma p-tau217 performance across two independent and clinically contrasting cohorts (a heterogeneous tertiary neurology population and a selectively characterized memory clinic cohort), providing one of the few external validations of LUMIPULSE-derived cutoffs in routine diagnostic settings. Second, we conducted sensitivity analyses of routine laboratory markers, including BUN and creatinine, offering evidence on the potential influence of common physiological variability on plasma p-tau217. Third, we evaluated both single- and two-cutoff frameworks and validated them externally, directly aligning our analyses with current guidelines and contributing to clinical implementation. Finally, the use of the fully automated LUMIPULSE platform highlights the assay’s scalability and clinical readiness, supporting its feasibility for broad implementation.

We acknowledge several limitations. Although the exploratory set was relatively large, it was drawn from a single center, which may limit generalizability to broader populations. Our analysis did not account for ethnicity or other sociodemographic variables, which could influence biomarker distributions. The clinical heterogeneity of our cohort, ranging from cognitive complaints to dementia, may also be viewed as a limitation; however, this was intentionally designed to reflect the diagnostic complexity of real-world memory clinics. The inclusion criteria were deliberately broad, potentially introducing variability related to comorbidities, which enhances external validity. Moreover, while we focus on plasma p-tau217 as a standalone biomarker, future studies should evaluate combined biomarker strategies (e.g., p-tau217/Aβ42 ratio) and extend the assessment of systemic influences. Longitudinal measures and more ethnically and geographically diverse samples will be essential for further refining the diagnostic and prognostic value of plasma p-tau217.

In conclusion, we demonstrated that plasma p-tau217, measured using the fully automated LUMIPULSE platform, outperformed p-tau181 in detecting AD pathology across clinically heterogeneous cohorts with cognitive complaints. The results presented in this work support plasma p-tau217 as a scalable and clinically robust blood-based biomarker with high utility for aiding diagnosis and patient management in AD.

## Methods

### Subjects

This study included individuals with cognitive complaints recruited from two distinct clinical contexts to reflect a broad range of diagnostic settings. One cohort (Coimbra) was drawn from a tertiary hospital setting with a wide spectrum of cognitive impairment presentations, while the other (Lisbon) came from a more selectively characterized memory clinic population. This design allowed us to evaluate the diagnostic performance of plasma p-tau biomarkers across real-world clinical environments and varying degrees of diagnostic complexity.

#### Coimbra cohort

The Coimbra cohort consisted of 494 individuals consecutively recruited at the Neurology Department of ULS Coimbra between January 2023 and December 2024. All participants were referred for a diagnostic evaluation of cognitive complaints and underwent a comprehensive clinical workup, which included a structured interview, physical and neurological examinations, and a brief neuropsychological assessment. CSF biomarkers for AD were obtained as part of routine diagnostic procedures. Additionally, peripheral blood samples were collected to assess plasma p-tau biomarkers and systemic variables relevant to potential physiological confounders.

Diagnostic categorization followed standardized criteria determined by their assistant neurologist, including subjective cognitive decline (SCD)^33^ (5%), mild cognitive impairment (MCI)^34^^34^ (56%), and dementia, defined as a “Major Cognitive Disorder” according to DSM-V criteria^35^ (37%). A small subset of individuals (2%) who did not meet the formal criteria for these categories but presented with clinically documented cognitive impairment were included in the analysis. These cases typically reflected atypical presentations or overlapping conditions, pending longitudinal clarification. This cohort was clinically diverse and representative of real-world tertiary care, covering a broad spectrum of cognitive decline severity. All individuals were clinically stable and free of acute medical conditions at the time of evaluation. In addition, data were collected on demographic information and routine blood parameters, including glucose, BUN, and creatinine, to explore sensitivity analyses and potential peripheral influences on plasma biomarker levels. Review of medical records confirmed that no individual met diagnostic criteria for chronic kidney disease (CKD); occasional isolated renal conditions (e.g., nephrolithiasis, prior nephrectomy) were present but did not reflect chronic renal dysfunction.

To support model development and testing, the Coimbra cohort was randomly divided into two main datasets: an exploratory set (80% of the sample, *n* = 395) and a validation set (20%, *n* = 99). The two sets did not differ in age, gender, cognitive impairment stage, or blood laboratory values.

#### Lisbon cohort

An independent sample of 100 participants was selected from the Cognitive Complaints Cohort (CCC), a prospective clinical cohort established at the Faculty of Medicine, University of Lisbon^32,36^. The CCC includes non-demented individuals with cognitive complaints who undergo a comprehensive and standardized neuropsychological evaluation, conducted by a team of trained neuropsychologists. Inclusion in the cohort requires the presence of subjective cognitive complaints and completion of a complete neuropsychological assessment. Participants are excluded if they have neurological or psychiatric disorders that could account for cognitive deficits (including major depression, as defined by DSM-IV-TR, or a Geriatric Depression Scale-15 score > 10), systemic illnesses with cerebral impact, substance abuse, or established dementia based on DSM-IV-TR criteria or MMSE scores below age- and education-adjusted cutoffs for the Portuguese population^37^.

All individuals included in the present study were recruited between 2000 and 2022, classified as having MCI, and had available CSF AD biomarker data. Participants were clinically stable and free from acute medical conditions at the time of assessment.

A subset of participants underwent amyloid PET imaging as part of their diagnostic workup. PET scans were acquired using ^11^C-Pittsburgh Compound B (^11^C-PiB PET) on a Philips PET/CT Gemini GXL scanner (ICNAS, University of Coimbra). Each scan was preceded by a low-dose CT for attenuation correction. PET images were classified as amyloid-positive or amyloid-negative by visual read, supported by a local support vector machine (SVM) classifier using voxel-wise grey-matter standardized uptake value ratios (SUVR) with cerebellar grey matter as reference.

### Laboratory determinations

#### CSF biomarkers determination

Samples from the two cohorts were collected as part of the patients’ routine clinical diagnostic examination. Standardized pre-analytical and analytical procedures were followed according to BIOMARKAPD guidelines for CSF-AD biomarkers^38^. Specifically, CSF samples were collected in sterile polypropylene tubes, immediately centrifuged at 1800 g for 10 min at 4 °C, aliquoted into 2 mL polypropylene tubes, and stored at -80 °C until analysis.

In Coimbra, CSF concentrations of Aβ42, Aβ40, t-tau, and p-tau181 were measured automatically and separately by commercially available immunoassays in the LUMIPULSE G600II platform (Fujirebio, Japan) at the Neurochemistry lab at ULS Coimbra, Portugal. In Lisbon, the same biomarkers were quantified using the LUMIPULSE G1200 platform (Fujirebio, Japan) at the Clinical Neurochemistry lab in Mölndal, Sweden. External quality control of the assays was performed under the scope of the Alzheimer’s Association Quality Control Program for CSF Biomarkers^39^.

To classify CSF data, we applied the AT(N) scheme^40^, which includes the Aβ42/40 ratio for assessing amyloid deposition (A), p-tau181 for evidence of Tau aggregation (T), and t-tau for neurodegeneration (N). For the Coimbra cohort, biomarker values were dichotomized as abnormal (+) or normal (-) using validated laboratory-specific cutoffs for the LUMIPULSE G600II platform^19^. Classification for the Lisbon cohort was based on established CSF thresholds^41^, and when available, amyloid status was confirmed by visual read of brain amyloid PET imaging. For individuals who also underwent PET, ^11^C-PiB PET served as a confirmatory measure. Concordance between CSF and PET was high, consistent with prior multicenter findings^42^. In rare instances of borderline CSF Aβ42/40 ratios, PET results provided definitive classification—no cases presented clearly conflicting CSF and PET amyloid status.

Based on the resulting AT(N) profiles, individuals were further stratified into three biologically defined categories: normal biomarkers (A-T-N-), AD continuum (A + T+/-N+/-), and non-AD pathological change (A-T+/-N+/-).

#### Peripheral blood-based biomarkers determination

Blood samples were collected into EDTA tubes on the same day as the lumbar puncture was performed. These samples were centrifuged at 1800 g (10 min at 4 °C), aliquoted into 2 mL polypropylene tubes, and stored at -80 °C until analysis.

Plasma concentrations of p-tau181 and p-tau217 for both cohorts were determined in the LUMIPULSE G600II platform (Fujirebio, Japan) at the Neurochemistry lab, ULS Coimbra. All samples were analyzed between 2023 and 2025. Quantification was done using Lumipulse^®^ G pTau 181 and Plasma Lumipulse^®^ G pTau 217 Plasma assays, following the manufacturer’s instructions. Concentrations were determined via a lot-specific calibration curve, assayed in duplicate, and quality control procedures were performed at the beginning of each test day to ensure that control values (low and high) fitted the target ranges.

### Statistical analysis

Statistical analysis was performed using R programming (version 4.5.0). A two-tailed p-value of less than 0.05 was considered statistically significant. To test for normal distribution, the Shapiro-Wilk test was used. As biomarker concentrations were not normally distributed, groups were compared using the Wilcoxon rank-sum test. Effect sizes between amyloid groups were quantified using Cliff’s delta, and differences in these values were evaluated using non-parametric bootstrap resampling (1000 iterations).

Associations between biomarkers were examined using Spearman’s rho. Correlation strength was assessed against conventional thresholds (very weak: 0.00-0.19, weak: 0.20–0.39, moderate: 0.40–0.59, strong: 0.60–0.79, and very strong: 0.80-1.00). Dependent correlations across CSF biomarkers were statistically compared using Steiger’s Z-test for overlapping correlations.

Diagnostic performance for amyloid positivity and AD-pathology was assessed using receiver operating characteristic (ROC) curves, with the area under the curve (AUC), sensitivity, specificity, positive and negative predictive values (PPV, NPV), Youden’s index, and accuracy calculated. To minimize variability related to sample partitioning, we generated 80/20 splits of the Coimbra cohort. We evaluated AUCs, Youden-based thresholds, 95% sensitivity thresholds, and the overall percentage of agreement (OPA) in the validation sets. From the five candidate splits, we selected the partition that achieved a stronger AUC and OPA, while maintaining the most balanced group distribution. ROC curves were compared using DeLong’s test. OPA was calculated as the sum of participants correctly classified by group over the total number of individuals.

For single cutoff analyses, optimal thresholds for p-tau181 and p-tau217 were calculated using Youden’s index. In addition, a two-cutoff framework was applied to p-tau217, with a high-sensitivity (95%) threshold and a high-specificity (95%) threshold. Values falling between these cutoffs were classified as intermediate.

Multivariate logistic regression was employed to assess predictors of amyloid positivity. The model presented was specified as: A (amyloid positivity) ~ plasma p-tau217 + age + BUN. Regression coefficients were exponentiated and reported as odds ratios (ORs) with 95% confidence intervals (CIs). Determinants of plasma p-tau217 concentration were evaluated using linear regression models. The primary model’s equation was plasma p-tau217 ~ BUN + age, and the secondary model’s was plasma p-tau217 ~ creatinine + age. Adjusted R^2^, unstandardized β-coefficients, and 95% CIs were reported for each model.

## Supplementary Information

Below is the link to the electronic supplementary material.

Supplementary Material 1

## Data Availability

The dataset used and/or analyzed during the current study are available from the corresponding author on reasonable request.
