# Supplementary material for: Plasma p-tau217, quantified by the fully automated LUMIPULSE G platform, outperforms p-tau181 in predicting amyloid pathology in cognitive complaints patients
Source: Sci Rep. 2026 Jan 15;16:4133. doi: 10.1038/s41598-025-34241-7 (PMC12858865; doi:10.1038/s41598-025-34241-7)
Supplement: Supplementary file 1 — Supplementary Material 1 [file 41598_2025_34241_MOESM1_ESM.pdf]

Content List of the Supplementary Material

Supplementary Table 1: Plasma p-tau181 and p-tau217 concentrations across the exploratory set following amyloid and tau pathology statuses with pairwise comparison.

Supplementary Table 2: Characterization of the exploratory set according to amyloid and tau pathology status.

Supplementary Table 3: Regression models evaluating a) prediction of amyloid status and b) predictors of plasma p-tau217 concentrations in the exploratory set.

Supplementary Figure 1: ROC curve analysis of plasma p-tau181 and p-tau217 for predicting the combination of amyloid and Tau status in the exploratory set.

Supplementary Figure 2: ROC analysis of pTau-based individual and combined predictors for amyloid status in the exploratory set.

.

Supplementary Table 1 - Plasma p-tau181 and p-tau217 concentrations across the exploratory set following amyloid and tau pathology statuses with pairwise comparison.

CSF AT Classification	Plasma p-tau181, pg/mL	Plasma p-tau181 Pairwise Comparison across groups	Plasma p-tau217, pg/mL	Plasma p-tau217 Pairwise Comparison across groups
A-T- (n=186)	1.06 (0.80-1.46)	vs **A-T+ (p=0.002)**, **A+T- (p<0.001)**, **A+T+ (p<0.001)**	0.09 (0.07-0.14)	vs **A-T+ (p<0.001)**, **A+T- (p=0.003)**, **A+T+ (p<0.001)**
A-T+ (n=30)	1.47 (1.21-1.90)	vs **A+T+ (p<0.001)**, A+T- (p=0.055)	0.13 (0.10-0.31)	vs **A+T+ (p<0.001)**, A+T- (p=0.96)
A+T- (n=29)	1.58 (0.93-1.96)	vs **A+T+ (p<0.001)**	0.25 (0.18-0.47)	vs **A+T+ (p<0.001)**
A+T+ (n=150)	2.40 (1.72-3.00)	-	0.65 (0.34-0.90)	-

Data is presented as median (25th-75th percentiles) or as a percentage. The categorization was done according to amyloid (A) and Tau pathology (T) status based on validated laboratory-specific CSF Aβ42/40 and p-tau181 cutoffs. Pairwise comparisons of plasma p-tau217 and p-tau181 concentrations across CSF AT classification groups. P-values were derived using Wilcoxon rank-sum tests with Bonferroni correction. Bold values indicate statistically significant differences ($p<0.05$).

Supplementary Table 2 – Characterization of the exploratory set according to amyloid and Tau pathology statuses.

	Total available (n)	A-T- (n = 186)	A+T+ (n = 150)	Combined (n = 336)	Test statistic
Demographic information					
Age, years	336	65.0 (59.0-70.0)	70.0 (64.5-74.0)	67.0 (61.0-73.0)	p<0.001
Sex, % females (n)	336	59% (110)	60% (90)	60% (200)	0.873
CSF biomarker concentrations					
CSF Aβ42/40 ratio	336	0.108 (0.102-0.115)	0.049 (0.043-0.056)	0.080 (0.051-0.109)	p<0.001
CSF p-tau181, pg/mL	336	29.1 (22.3-37.0)	101.1 (78.4-133.7)	44.9 (27.8-94.6)	p<0.001
CSF t-tau, pg/mL	336	230.5 (174.0-292.8)	615.5 (466.5-828.2)	329.0 (217.0-584.5)	p<0.001
Blood-based biomarker concentrations					
Plasma p-tau181, pg/mL	336	1.06 (0.80-1.46)	2.40 (1.72-3.00)	1.51 (1.01-2.45)	p<0.001
Plasma p-tau217, pg/mL	336	0.09 (0.07-0.14)	0.65 (0.34-0.90)	0.19 (0.09-0.62)	p<0.001
Glucose, mg/dL	311	94.0 (84.0-110.0)	93.0 (85.0-108.5)	94.0 (84.5-109.0)	0.352
Creatinine, mg/dL	308	0.76 (0.68-0.88)	0.77 (0.70-0.85)	0.76 (0.69-0.86)	0.785
Blood Urea Nitrogen, mg/dL	312	16.0 (13.6-19.4)	17.0 (13.7-20.5)	16.4 (13.7-20.0)	0.218

Data is presented as median (25th-75th percentiles) or as a percentage. The categorization was done according to amyloid (A) and Tau pathology (T) status based on validated laboratory-specific CSF Aβ42/40 and p-tau181 cutoffs. Tests used: Wilcoxon rank-sum for continuous variables and Pearson's chi-squared for the nominal category. Abbreviations: Aβ = amyloid beta; CSF = cerebrospinal fluid; n = count or number of individuals per variable; p-tau181 = phosphorylated Tau protein in position 181; p-tau217 = phosphorylated Tau protein in position 217; t-tau = total Tau protein.

Supplementary Table 3: Regression models evaluating a) prediction of amyloid status and b) predictors of plasma p-tau217 concentrations in the exploratory set.

Panel A – Logistic regression predicting amyloid positivity (A+ vs A-)

Predictor	β (Estimate)	Odds ratio (OR)	95% Confidence Interval (OR)	p-value
Intercept	0.000124	-	-	<0.001
Plasma p-tau217 (pg/mL)	4.61	100.36	36.45-317.33	<0.001
Age (years)	0.12	1.13	1.08-1.18	<0.001
Blood urea nitrogen (mg/dL)	-0.046	0.95	0.91-1.00	0.046

Panel B – Linear regression predicting plasma p-tau217 concentrations

Model 1 (Age + BUN)

Predictor	β (Estimate)	95% CI	p-value
Intercept	-0.095	-0.48 to 0.29	0.627
Blood urea nitrogen (mg/dL)	0.009	0.001 to 0.017	0.026
Age (years)	0.005	-0.001 to 0.011	0.090

Adjusted R^2 = 0.020
Residual standard error = 0.450
F-statistic = 4.75, df = 2,357, p = 0.009

Model 2 (Age + Creatinine)

Predictor	β (Estimate)	95% CI	p-value
Intercept	-0.029	-0.44 to 0.38	0.381
Creatinine (mg/dL)	0.030	0.278 to 0.244	0.244
Age (years)	0.006	0.000 to 0.012	0.041

Adjusted R^2 = 0.007
Residual standard error = 0.454
F-statistic = 2.194, df = 354, p = 0.113

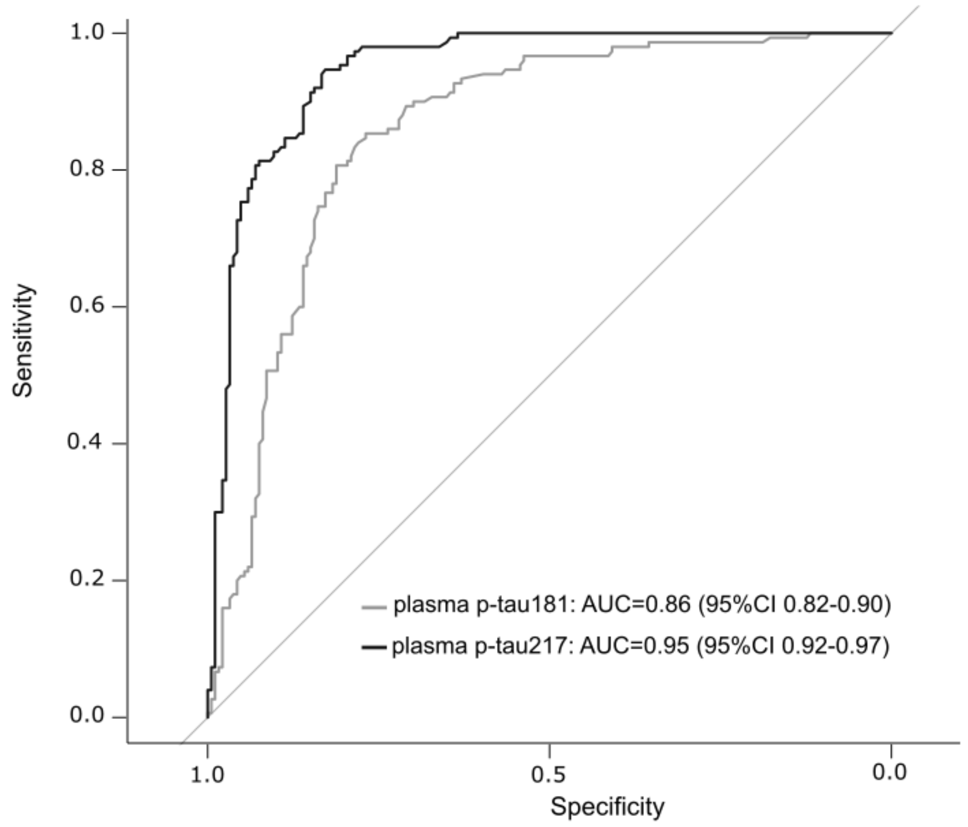

Biomarker	Threshold	Sensitivity	Specificity	NPV	PPV	Youden	Accuracy
Plasma p-tau181	1.50 pg/mL	0.85	0.77	0.87	0.75	0.62	0.81
Plasma p-tau217	0.17 pg/mL	0.95	0.82	0.95	0.81	0.77	0.88

Supplementary Figure 1: **ROC curve analysis of plasma p-tau181 and p-tau217 for predicting the combination of amyloid and Tau status in the exploratory set.** Receiver operating characteristic (ROC) curves illustrate the diagnostic performance of plasma p-tau181 (grey curve) and p-tau217 (black curve) in distinguishing amyloid- and phosphorylated Tau-positivity (A+T+; n=150) from amyloid- and phosphorylated Tau-negative (A-T-; n=186) individuals within the exploratory set (n=395). The area under the curve (AUC) is presented with 95% confidence intervals (CI). The table below the graph presents performance metrics for each analyte, including optimal threshold, sensitivity, specificity, positive predictive value (PPV), negative predictive value (NPV), Youden's index, and overall accuracy.

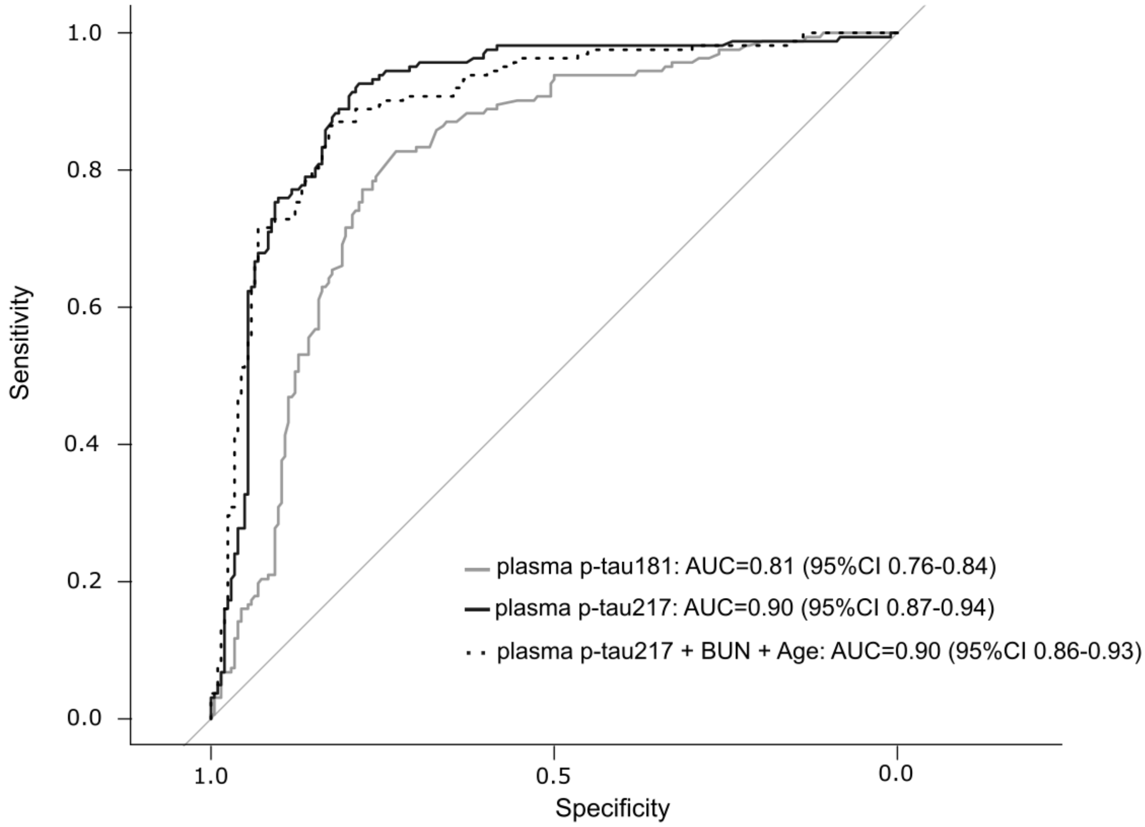

Supplementary Figure 2: ROC analysis of pTau-based individual and combined predictors for amyloid status in the exploratory set. Receiver operating characteristic (ROC) curves illustrate the diagnostic performance of plasma p-tau181 (grey curve) and p-tau217 (black curve), and a multivariate logistic regression model combining p-tau217, blood urea nitrogen (BUN), and age (dashed curve) for predicting amyloid status in the exploratory set (n=366; A-, n=204; A+, n=162). The area under the curve (AUC) is presented with 95% confidence intervals (CI).
